# Activity Subspaces in Medial Prefrontal Cortex Distinguish States of the World

**DOI:** 10.1523/JNEUROSCI.1412-21.2022

**Published:** 2022-05-18

**Authors:** Silvia Maggi, Mark D. Humphries

**Affiliations:** School of Psychology, University of Nottingham, Nottingham NG7 2RD, United Kingdom

**Keywords:** decoding, learning, medial prefrontal cortex, population activity, sleep reactivation

## Abstract

Medial prefrontal cortex (mPfC) activity represents information about the state of the world, including present behavior, such as decisions, and the immediate past, such as short-term memory. Unknown is whether information about different states of the world are represented in the same mPfC neural population and, if so, how they are kept distinct. To address this, we analyze here mPfC population activity of male rats learning rules in a Y-maze, with self-initiated choice trials to an arm end followed by a self-paced return during the intertrial interval (ITI). We find that trial and ITI population activity from the same population fall into different low-dimensional subspaces. These subspaces encode different states of the world: multiple features of the task can be decoded from both trial and ITI activity, but the decoding axes for the same feature are roughly orthogonal between the two task phases, and the decodings are predominantly of features of the present during the trial but features of the preceding trial during the ITI. These subspace distinctions are carried forward into sleep, where population activity is preferentially reactivated in post-training sleep but differently for activity from the trial and ITI subspaces. Our results suggest that the problem of interference when representing different states of the world is solved in mPfC by population activity occupying different subspaces for the world states, which can be independently decoded by downstream targets and independently addressed by upstream inputs.

**SIGNIFICANCE STATEMENT** Activity in the medial prefrontal cortex plays a role in representing the current and past states of the world. We show that during a maze task, the activity of a single population in medial prefrontal cortex represents at least two different states of the world. These representations were sequential and sufficiently distinct that a downstream population could separately read out either state from that activity. Moreover, the activity representing different states is differently reactivated in sleep. Different world states can thus be represented in the same medial prefrontal cortex population but in such a way that prevents potentially catastrophic interference between them.

## Introduction

The medial prefrontal cortex (mPfC) plays key roles in adaptive behavior, including reshaping behavior in response to changes in a dynamic environment (Euston et al., 2012) and in response to errors in performance (Narayanan and Laubach, 2008; Laubach et al., 2015). Damage to mPfC prevents shifting behavioral strategies when the environment changes (Laskowski et al., 2016; Guise and Shapiro, 2017). Single neurons in mPfC shift the timing of spikes relative to hippocampal theta rhythms just before acquiring a new action–outcome rule (Benchenane et al., 2010). And multiple labs have reported that global shifts in mPfC population activity precede switching between behavioral strategies (Rich and Shapiro, 2009; Durstewitz et al., 2010; Karlsson et al., 2012; Powell and Redish, 2016) and the extinction of learned associations (Russo et al., 2021).

Adapting behavior depends on knowledge of both the past and the present state of the world. Deep lines of research have established that mPfC activity represents information about both. The memory of the immediate past is maintained by mPfC activity in tasks requiring explicit use of working memory (Baeg et al., 2003; Fujisawa et al., 2008; Spellman et al., 2015). The use of such memory is seen in both the impairment arising from mPfC lesions (Rich and Shapiro, 2007; Young and Shapiro, 2009; Laskowski et al., 2016) and the role of mPfC in error monitoring (Laubach et al., 2015). Representations of stimuli and features happening in the present have been reported in a variety of decision-making tasks throughout PfC (Averbeck et al., 2006; Rigotti et al., 2013; Hanks et al., 2015; Siegel et al., 2015) and specifically within rodent mPfC (Sul et al., 2010; Ito et al., 2015; Guise and Shapiro, 2017).

Little is known though about how mPfC activity represents multiple states of the world. Prior studies have shown that past and upcoming choices can both modulate activity of neurons in the same mPfC population (Baeg et al., 2003; Ito et al., 2015), but none have compared how different states of the world are represented. Thus important questions remain regarding if and how different world states are encoded in the same mPfC population and how those representations are kept distinct.

To address these questions, we reanalyze here mPfC population activity from rats learning new rules on a Y-maze (Peyrache et al., 2009). This task had distinct trial and intertrial interval phases, and we have previously shown that task features of the preceding trial can be decoded from the population activity in the intertrial interval (Maggi et al., 2018), showing that mPfC activity in this task depends on the state of the world. We can thus address our key questions here by asking whether population activity in the trials also represents the state of the same task features and, if so, how that representation is kept distinct between the trial and intertrial interval phases.

We find that the mPfC population activity occupies different subspaces between trials and intertrial intervals, providing a basis for separately representing at least these two distinct states of the world. Consistent with representing world states, task features could be decoded from activity in both the trial and intertrial interval phases but were strongly distinct: decoding was of the present features in the trial and predominately of features of the preceding trial during the intertrial interval. Decoding axes were, or close to, orthogonal between the trials and intertrial intervals, showing that the subspaces supported distinct encodings. Further, population activity of the trials and intertrial intervals preferentially reactivated in post-training sleep in different ways: preferential reactivation of trial activity uniquely occurred after learning and correlated with performance during training. Our results thus suggest that representing different world states using independently decodable axes within a mPfC population could prevent interference between them, allowing them to be separately accessed by both downstream and upstream populations.

## Materials and Methods

### Task description and electrophysiological data

All data in this study come from previously published data (Peyrache et al., 2009). Full details of training, spike sorting, and histology can be found in Peyrache et al. (2009). The experiments were conducted in accordance with institutional (Centre National de la Recherche Scientific Comité Opérationnel d'Éthique dans les Sciences de la Vie) and international (National Institutes of Health guidelines) standards and legal regulations (Certificate no. 7186, French Ministère de l'Agriculture et de la Pêche) regarding the use and care of animals.

**Figure 1.**
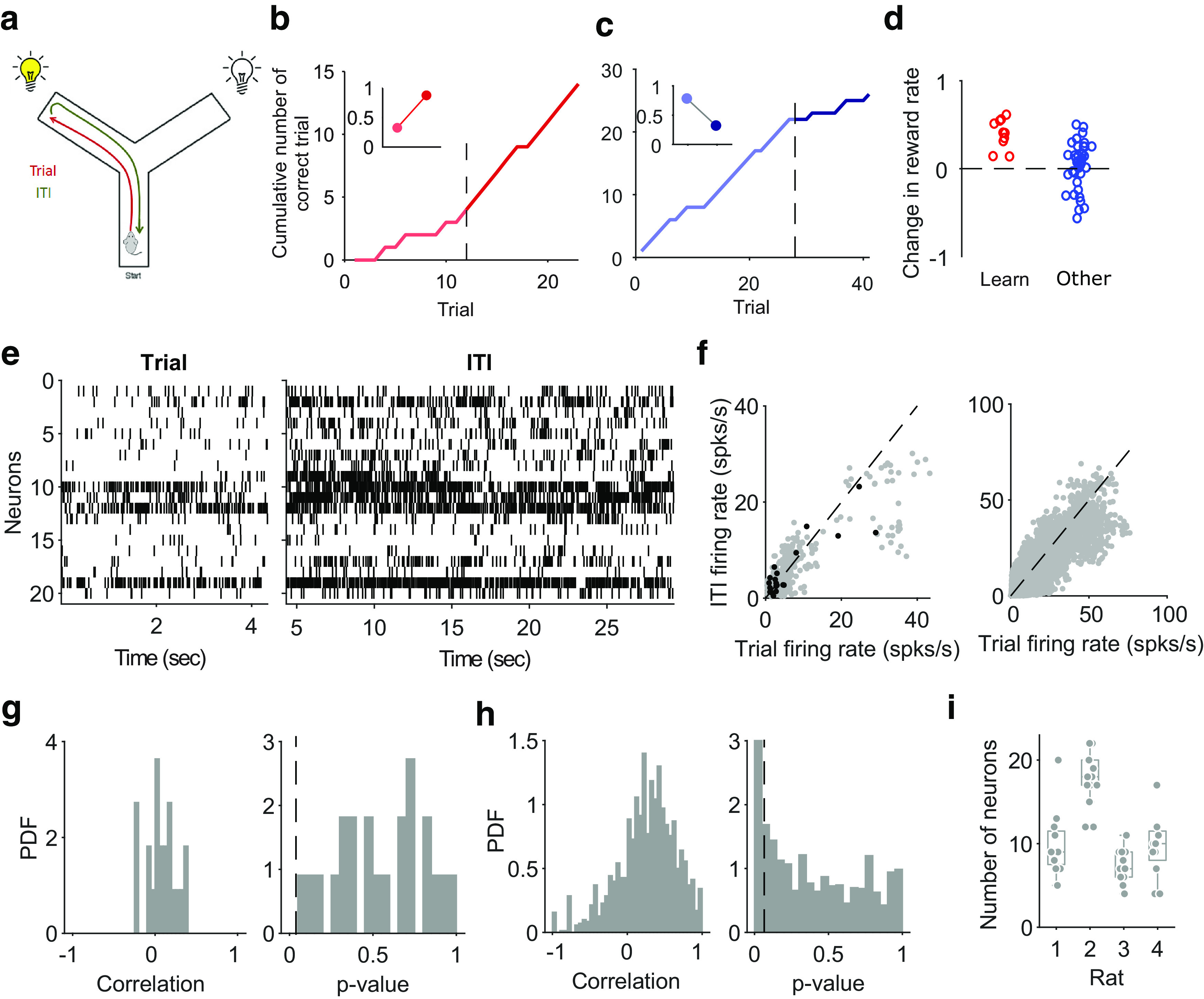
Rule learning and neural activity on the Y-maze task. ***a***, Schematic of the Y-maze task, showing a rat at the start position. A trial is the period from the start position to the end of the chosen arm; the intertrial interval is the return from the arm end to the start position. On each trial one arm end was lit, chosen in a pseudorandom order, regardless of whether it was relevant to the currently enforced rule. Across sessions, animals were asked to learn one of the following four rules in the sequence: Go to the right arm, go to the lit arm, go to the left arm, go to the dark arm. Rules switched after 10 correct choices (or 11 of 12). ***b***, Example reward curve from a learning session, plotting the cumulative number of correct trials. Black dashed line identifies the learning trial as the first of three consecutive correct trials followed by at least 80% correct trials. Inset, Reward rates before (light red) and after (dark red) the learning trial. Reward rates were given by the slope of linear regressions fitted to the reward curve before and after the learning trial. ***c***, Example reward curve from an Other session, one of eight in which the rule switched; the black dashed line identifies the rule change trial. Inset, Reward rates before and after the rule change. ***d***, Change in reward rate during all learning sessions (red) or Other sessions (blue). ***e***, Raster plots of spiking activity in the medial prefrontal cortex during a single trial and the following intertrial interval. ***f***, Neuron firing rates in each trial and in the following intertrial interval. Left, An example session, black dots are the data in ***e***. Right, All sessions. ***g***, For the same example session, the distribution of Spearman's rank coefficients between the population vectors of firing rates in the trial and intertrial interval (left) and the corresponding *p* values (right); *p* = 0.05 is indicated by the dashed line. ***h***, As in ***g*** for all sessions. ***i***, The number of neurons in populations analyzed here, by rat. Each symbol is a session. Boxes plot median and interquartile range (IQR); whiskers extend to 1.5 IQR.

Four male Long-Evans rats were implanted with tetrodes in the medial wall of prefrontal cortex, covering the prelimbic and infralimbic regions, and trained on a Y-maze task (Fig. 1*a*). During each session, neural activity was recorded for 20–30 min of sleep or rest epoch before the training epoch, in which rats worked at the task for 20–40 min. After that, another 20–30 min of sleep or rest epoch recording followed. During the sleep epochs, intervals of slow-wave sleep were identified off-line from the local field potential (Peyrache et al., 2009; Benchenane et al., 2010).

The Y-maze had symmetrical arms, 85 cm long, 8 cm wide, and separated by 120°, connected to a central circular platform (denoted as the choice point throughout). The two choice arms had a light at the end, one of which was lit during each trial in a pseudorandom sequence. Rats self-initiated a trial by leaving the beginning of the start arm. A trial finished when the rat reached the end of the chosen goal arm. If the chosen arm was correct according to the current rule, the rat was rewarded with drops of flavored milk. As soon as the animal reached the end of the chosen arm, an intertrial interval started and lasted until the rat completed its self-paced return to the beginning of the start arm. The central platform was raised once the rat passed it to prevent backtracking along the choice arms. The light was extinguished during the return journey; unfortunately from the data available to us it is not clear exactly when (F. Battaglia, personal communication).

Each rat was exposed to the task completely naive and had to learn each rule by trial and error. The rules were presented in the following sequence: Go to the right arm, go to the lit arm, go to the left arm, go to the dark arm. A rule was switched to the next in the sequence when the animal had achieved 10 correct trials in a row, or 11 of 12. Across the four rats, there were eight rule switches in total.

The recording sessions taken from the study of Peyrache et al. (2009) were 53 in total. Each of the four rats learned at least two rules, and they respectively contributed to 14, 14, 11, and 14 sessions. We used 49 of these sessions for our analysis, of between 7 and 51 trials each. One session was omitted for missing position data, one for consistent choice of the right arm (in a dark arm rule) preventing decoder analyses (see below), and one for missing spike data in a few trials. An additional session was excluded for having only two neurons firing in all trials. Tetrode recordings were spike sorted within each recording session. Spikes were recorded with a resolution of 0.1 ms. Simultaneous tracking of the position of the rat was recorded at 30 Hz.

### Testing for separable population activity

We evaluated the difference between population activity in the trial and intertrial intervals of a session by quantifying their separability in a low-dimensional space. For consistency with our previous work, for each session we selected the *N* active neurons that fired at least one spike on each trial (Fig. 1*e*), allowing us to directly compare the decoding results obtained here (see below) with those in Maggi et al. (2018); the populations thus ranged between 4 and 22 neurons (Fig. 1*i*).

We used principal component analysis (PCA) to project the population vectors of a session onto a common set of dimensions. For each session, we constructed an *N-*length vector of neuron firing rates in each trial $r_{t}$, resulting in the set of population firing rate vectors $\left\{r_{t}(1),...,r_{t}(T)\right\}$ across the *T* trials of a session. We similarly constructed the set of vectors of neuron firing in each intertrial interval $\left\{r_{I}(1),...,r_{I}(T)\right\}$. We then constructed the data matrix **X** from the firing rate vectors of the population, by concatenating trials and intertrial intervals in their temporal order ${\left\{r_{t}(1),r_{I}(1),...,r_{t}(T),r_{I}(T)\right\}}^{\text{′}}$ across the *T* trials of a session; the resulting matrix thus had dimensions of 2*T* rows and *N* (neurons) columns. Applying PCA to **X**, we projected the firing rate vectors on to the top *d* principal axes (the eigenvectors of $X^{\prime }X$) to create the top *d* principal components. For each set of *d* components, we quantified the separation between the projected trial and intertrial interval population vectors using a linear classifier (support vector machine), and report the proportion of misclassified vectors. We repeated this for between *d* = 1 and *d* = 4 axes for each session.

### Linear decoding of task features

To predict which task feature was encoded in mPfC population activity, we trained and tested a range of linear decoders (Hastie et al., 2009). Here, we report the results obtained using a logistic regression classifier, but for robustness we also tested three other decoders—linear discriminant analysis, linear support vector machines, and a nearest neighbors classifier—and found similar results. The full details of the decoding analysis can be found in Maggi et al. (2018).

Task information of each trial was binary labeled for three features, outcome (labels 0, 1), the direction of the chosen arm (labels left, right), and the arm position of the light cue (labels left, right). We used leave-one-out cross-validation to decode each feature from population activity, holding out the vector of the *i*th trial, $r_{t}(i)$, training the classifier on the *N* – 1 remaining trial vectors, and then using the resulting weight vector to predict the label of the feature for the held-out trial. We quantified the accuracy of the decoder as the proportion of correctly predicted labels over all *T* held-out trials of a session. The same approach was used for the intertrial intervals, using the set of firing rate vectors $\left\{r_{I}(1),...,r_{I}(T)\right\}$ across the *T* intertrial intervals.

For decoding at different positions in the maze, we first linearized the maze, divided it into five equally sized sections, and then computed the *N-*length firing rate vector of the population for each position *p*, $r^{p}{}_{t}$ in the trial. For each trial *t* = 1,…,*T* of a session and each section of the maze *p* = 1,…,5, the set of population firing rate vectors $\left\{r^{p}{}_{t}(1),...,r^{p}{}_{t}(T)\right\}$ was used to train the cross-validated decoder. The same approach was used for the position-dependent vectors $\left\{r^{p}{}_{I}(1),...,r^{p}{}_{I}(T)\right\}$ in the intertrial intervals of a session.

For each rat and each session, the distribution of outcomes and arm choices depended on the performance of the rats, which could differ from 50%. Therefore, we trained and cross-validated the same classifier on the same datasets but shuffling the labels of the task features across trials. In this way we obtained the accuracy of detecting the correct labels by chance. We repeated the shuffling and fitting 50 times, and we averaged the accuracy across the 50 repetitions.

### Testing for independent decoding

To compare the decoding axes between the trials and intertrial intervals, we again trained the classifier separately for each of the three task features but now using all the population firing rate vectors of a session, first for the trials $\left\{r_{t}(1),...,r_{t}(T)\right\}$ and then for the intertrial intervals $\left\{r_{I}(1),...,r_{I}(T)\right\}$. For a given feature *f*, we then computed the angle, θ*_f_*, between the resulting vectors of decoding weights for the trials, *w_t_*(*f*), and intertrial intervals, *w_I_*(*f*), as follows: $\theta =\cos^{-1}\left(\frac{w_{t}(f)\cdot w_{I}(f)}{\left||w_{t}(f)||||w_{I}(f)|\right|}\right)$. Similarly, to assess how the different features were simultaneously encoded during a task phase, we computed the angles between the decoding vectors of two features *f*_1_ and *f*_2_ within a trial, or within an intertrial interval.

We evaluated the degree of independence between the trial and intertrial interval decoding by attempting to cross-decode a task feature in one phase from the activity in the other. For a given task feature, we took the above classifier trained on all trials of a session and tested its decoding on all intertrial intervals of the same session, with performance reported as the percentage of correctly labeled intertrial intervals. We also tested this in reverse, decoding the feature in the trials from the classifier trained to decode the same feature from all the intertrial intervals. To check we were not overfitting when using a decoder trained on all *T* phases, we further tested cross-decoding using leave-one-out by leaving out the *i*th trial interval pair, training on *N* – 1 trials, and predicting the *i*th intertrial interval (and vice versa for training on intertrial intervals and testing on trials). Performance for leave-one-out cross-decoding was reported as the percentage of correctly labeled held-out trials (or intertrial intervals) over all trials of the session.

### Behavioral analysis

To check whether our decoding results depended on potentially different behaviors or task demands, we divided the sessions in two different ways, by rule type and by learning type. For the rule type, we grouped sessions by whether the target rule was a direction-based rule (so putatively egocentric) or a cue-based rule (so putatively allocentric).

To group by learning type, we identified learning sessions according to the criteria of the original study (Peyrache et al., 2009) of a session with three consecutive correct trials followed by a performance of at least 80% correct. The first of the three correct trials was the learning trial. Only 10 sessions satisfied these criteria. All sessions that did not meet these criteria were labeled Other.

We quantified performance in learning sessions by fitting a piecewise linear regression model to the cumulative reward curve, using robust regression to fit lines before and after the learning trial. The slopes of the two lines gave us the rate of reward accumulation before (*r_before_*) and after (*r_after_*) the learning trial (Fig. 1*b*). We quantified performance on all other sessions in a similar way (Fig. 1*c*,*d*). For the eight rule-change sessions, we considered the slopes of the regression lines before and after the rule-switch trial. For all remaining sessions, we looked for any performance change by fitting the piecewise linear regression model to each trial in turn (allowing a minimum of five trials before and after each tested trial). We then found the trial at which the increase in slope ($r_{after}\;-\;r_{before}$) was maximized, indicating the point of steepest inflection in the cumulative reward curve.

### Reactivation of task-feature representations in sleep

**Figure 2.**
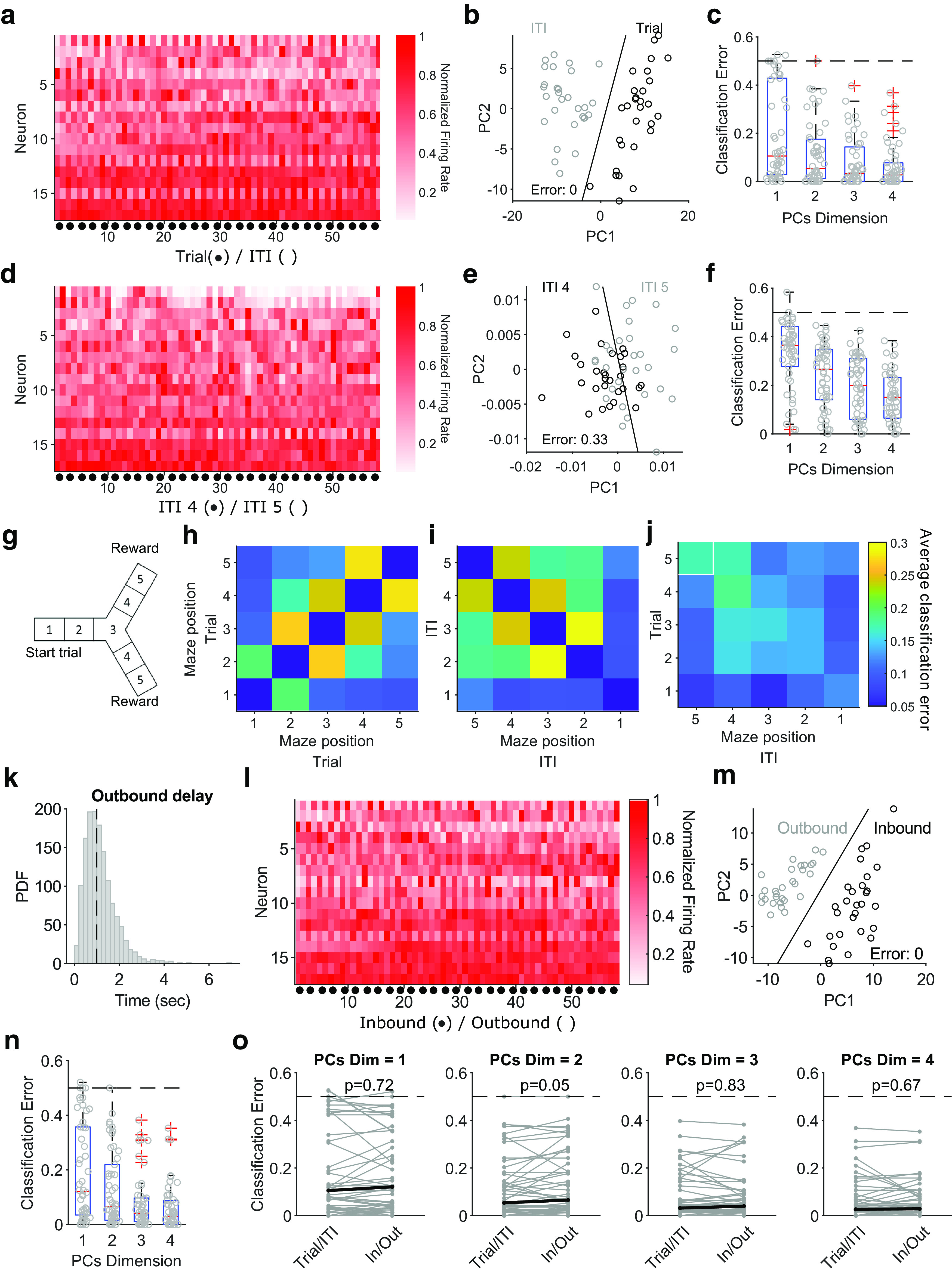
Population activity is easily separable between trials and intertrial intervals. ***a***, Population activity vectors for the trials (black dot) and following intertrial intervals of one session with 54 trial-ITI pairs. The heat map shows the normalized firing rate for each neuron. ***b***, Projection of population activity vectors of that session on to two dimensions shows a complete separation of trial and intertrial interval activity. The black line is the linear separation found by the classifier. PC, Principal component. ***c***, Summary of classification error over all sessions as a function of the number of dimensions. Each gray dot is the error for one session at that number of projecting dimensions. Dashed line gives chance performance. Box plots show medians (red line), interquartile ranges (blue box), and outliers (red pluses). ***d***–***f***, Same as ***a–c*** but comparing population activity vectors for maze sections 4 and 5 in the intertrial interval. ***g***, Schematic of maze sections. ***h***, The average classification error in the separation of population activity vectors between each pair of maze sections within trials. Classification error is for projections of the population activity vectors in a two-dimensional space. ***i***, As for ***h***, within intertrial intervals. ***j***, As for ***h***, for the separability of the trial and intertrial interval population activity across maze sections. The white square indicates the arm-end position, where the transition from trial to intertrial interval occurs. ***k***, Distribution of the delay between the start of the intertrial interval and the start of the outbound phase, with a median delay of 1 s (dashed line) and a mean of 1.14 s. ***l***, Same session as ***a*** but divided by inbound and outbound phases. ***m***, As for ***b***, classifying vectors by inbound and outbound phases. ***n***, As for ***c***, for inbound and outbound vectors. ***o***, Comparing the classification error for the population vectors divided by trial-ITI or by inbound-outbound phases. Each line is a session; black lines show medians. The *p* values are from paired Wilcoxon signed-rank tests.

To quantify the reactivation of waking activity in pre-training and post-training sleep, we used the population firing rate vectors computed for the decoder {r(1),...,r(T)}. We considered here the average population vector for a feature in each session, computed across all the trials for each feature. For example, we quantified the average population firing rate vector for all the right-choice trials and separately for all the left-choice trials. This vector thus represented the region in the activity subspace (Fig. 2) occupied by that particular feature in the trial or the intertrial interval.

We then compared this feature-specific activity vector with the firing rate vector of each 1 s time bin of slow-wave sleep pre-training and post-training using Spearman's correlation coefficient. This gave us a distribution of correlations between the feature-specific vector and the population activity vectors during pre-training slow-wave sleep and a distribution of correlations between the feature-specific vector and the population activity vectors during post-training slow-wave sleep.

Spearman's coefficient was chosen specifically to compare the relative activity of the neurons in the population between training and sleep epochs, and so we call this “reactivation,” not “replay.” Replay implies that specific patterns of firing from waking, such as sequences of place cells (Skaggs and McNaughton, 1996; Lee and Wilson, 2002; O'Neill et al., 2008; Denovellis et al., 2021) or sequences of neurons in an ensemble (Euston et al., 2007; Peyrache et al., 2009, 2010), reappear during sleep or quiescence. As we use it here, reactivation is assessing how well the sleep activity aligns with the two subspaces of trial and intertrial interval activity during training, and consequently suggests whether those two are being revisited.

If a feature-specific activity vector was preferentially reactivated in post-training sleep, then we would expect the distribution of the correlation coefficients between a feature and post-training slow-wave sleep to be right shifted compared with the distribution of the correlation coefficients between the same feature and pre-training slow-wave sleep. We quantified this shift by measuring the difference in the medians $\left(M_{post}\;-\;M_{pre}\right)$ between the two distributions of correlation coefficients. If the difference was positive, then we had a higher correlation of the feature-specific vector with the activity in post-training slow-wave sleep than with the activity in pre-training slow-wave sleep. If negative, then the feature-specific vector was more similar to the pre-training slow-wave sleep population activity.

To control for different time scales of reactivation in sleep we repeated the same procedure, changing the time bin in the slow-wave sleep pre-training and post-training. Bin sizes from 100 ms to 10 s were chosen to range below and above the mean length of a trial (∼6.5 s).

### Statistics

**Figure 3.**
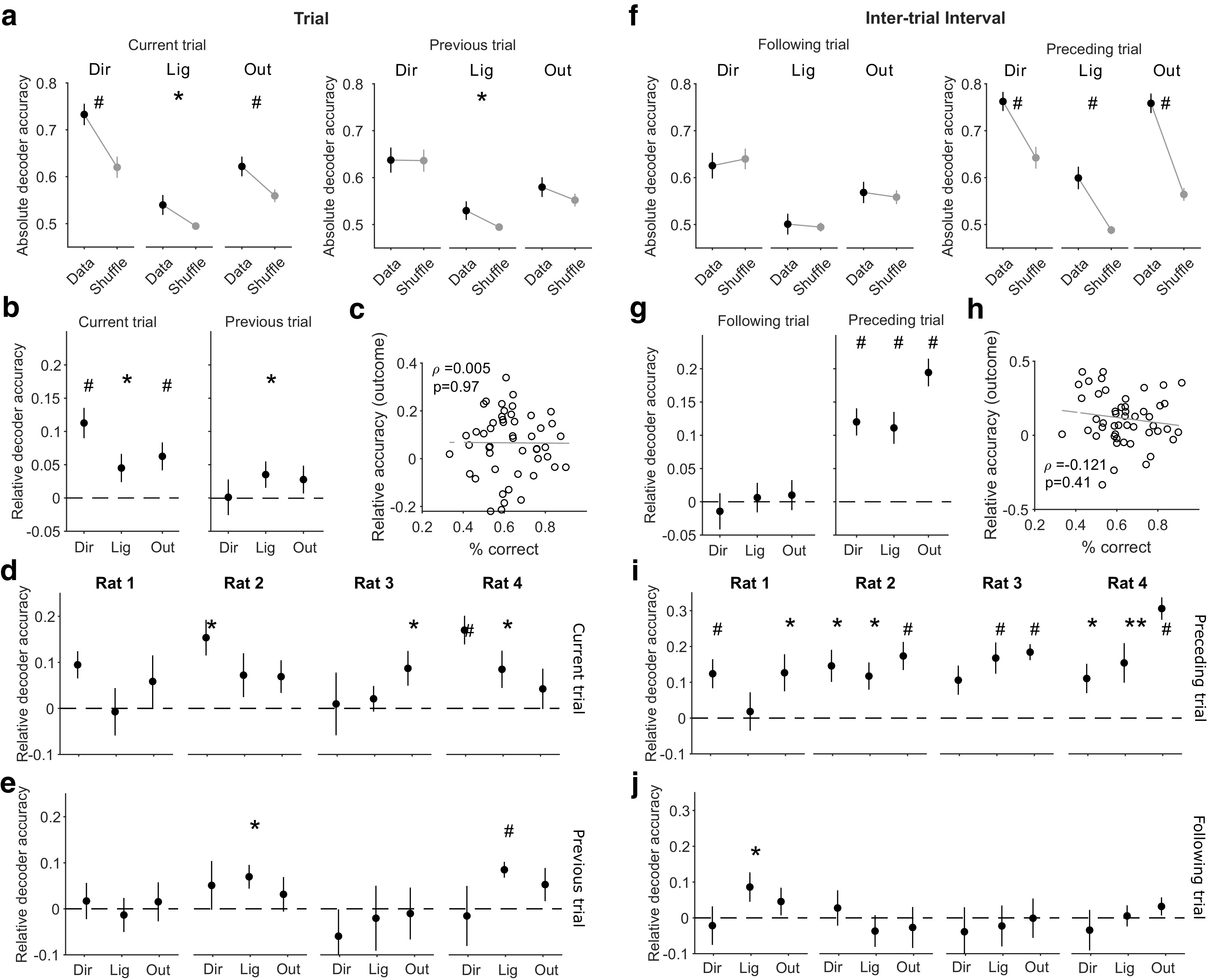
Decoding of different task states in the trials and intertrial intervals. ***a***, Accuracy of decoding task features from population activity during trials. In black we plot the accuracy of decoding the choice of arm direction (Dir), light position (Lig), and outcome (Out) for the current trial (left) and the previous trial (right). In gray we plot the decoding accuracy of shuffled labels across trials. Differences assessed using a paired Wilcoxon signed-rank test, *p < 0.05, **p < 0.01, #*p* < 0.005. Symbols plot means ± SEM across 49 sessions. ***b***, As for ***a***, plotted as relative decoding accuracy, the difference between the decoding accuracy of the data and of the mean of the shuffled data in that session. All *p* values are given for a Wilcoxon signed-rank test against zero median. ***c***, No correlation between session performance and the accuracy of decoding the upcoming outcome of a trial. Gray line is a linear regression fit to the data. ***d***, ***e***, Per-subject breakdown of the decoding results in ***b***. ***f***, As for ***a***, but for population activity during the intertrial intervals of each session, decoding the features of the following (left) and preceding (right) trials. ***g***, as for ***b***, for decoding during the intertrial interval. ***h***, As for ***c***, for decoding the outcome preceding the intertrial interval. ***i***, ***j***, Per-subject breakdown of the decoding results in ***g***.

**Figure 4.**
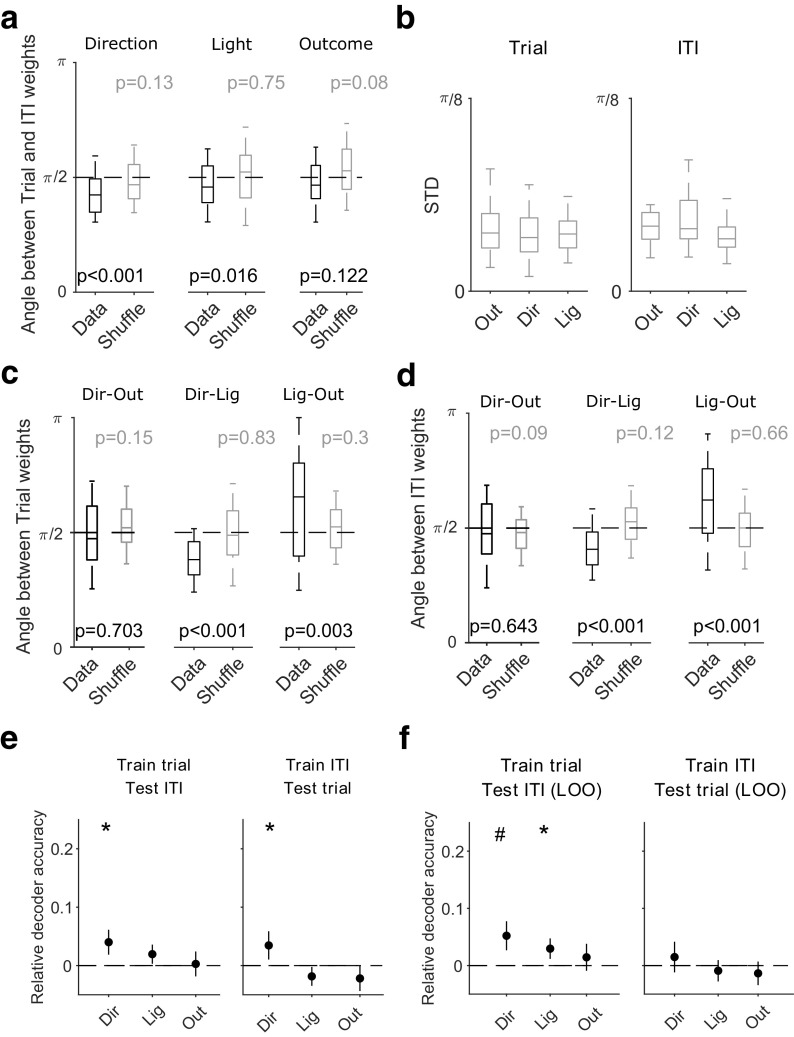
Independent decoding of task features between trials and intertrial intervals. ***a***, Angles between the decoding weight vectors for trials and intertrial intervals for each task feature. For reference, we also compute the angle between trial and intertrial interval decoding vectors obtained by training on shuffled label data (gray). Box plots show median (line), interquartile range (box), and 95% interval (tails) over all sessions. The *p* values are from Wilcoxon rank sum tests for the difference from π/2. ***b***, Variation of angles between decoding axes within the same task phase. We plot here the average standard deviation (STD) of the angles between decoding axes for the same feature in either the trials (left) or intertrial intervals (right) of a session. Within-session decoding axes for each feature were taken from the classifiers fitted to each training subset of trials or intertrial intervals during the cross-validation. ***c***, As for ***a***, but comparing the decoding weight vectors between features within trials. ***d***, As for ***c***, within intertrial intervals. ***e***, Cross-decoding performance for each task feature of the current trial, using the decoding vectors in ***a***. Left, Performance when the decoder was trained on activity during trials and tested on activity in the intertrial intervals. Black dashed line shows the chance level obtained training the classifier on shuffled labels for the trials and testing on intertrial intervals given the same shuffled labels. Right, Performance when the decoder was trained on activity from the intertrial intervals, and tested on activity in the trials; *p < 0.05, **p < 0.01, #*p* < 0.005. ***f***, As for ***e***, using leave-one-out cross-decoding.

**Figure 5.**
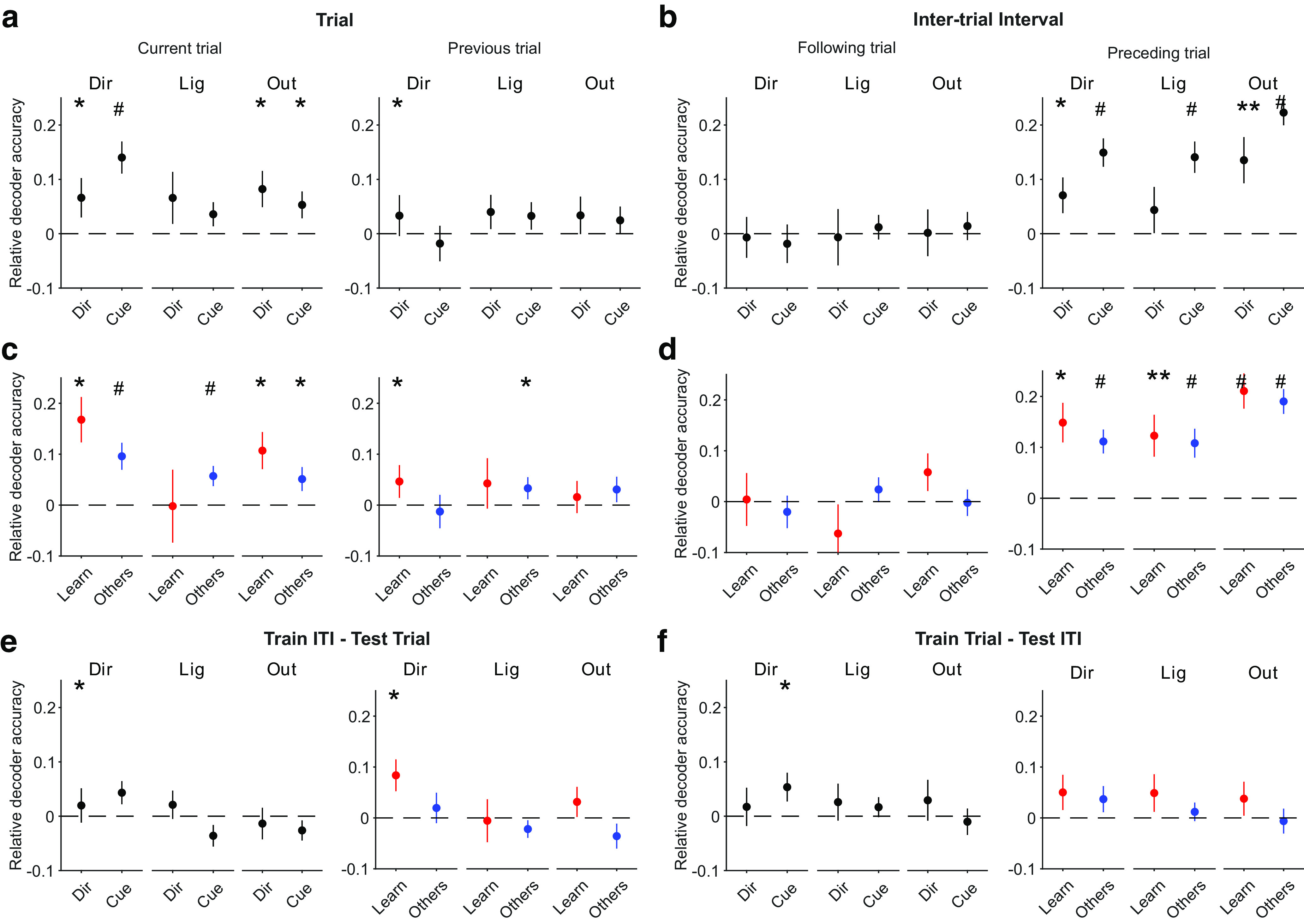
Breakdown of decoding and cross-decoding by session type. ***a***, Breakdown of the trial decoding results in Figure 3*b* by the rule type of each session (15 direction rule sessions, 34 cue rule sessions); *p < 0.05, **p < 0.01, #*p* < 0.005. ***b***, As for ***a***, breakdown of the intertrial interval decoding results by the rule type of each session. ***c***, Breakdown of the trial decoding results in Figure 3*b* by whether a rule was learned in a session (10 identified learning sessions, 39 other sessions). ***d***, As for ***c***, breakdown of the intertrial interval decoding results by learning and other sessions. ***e***, ***f***, Breakdown of the cross-decoding results in Figure 4*d* by rule type (left) and performance type (right) for each cross-decoding direction.

**Figure 6.**
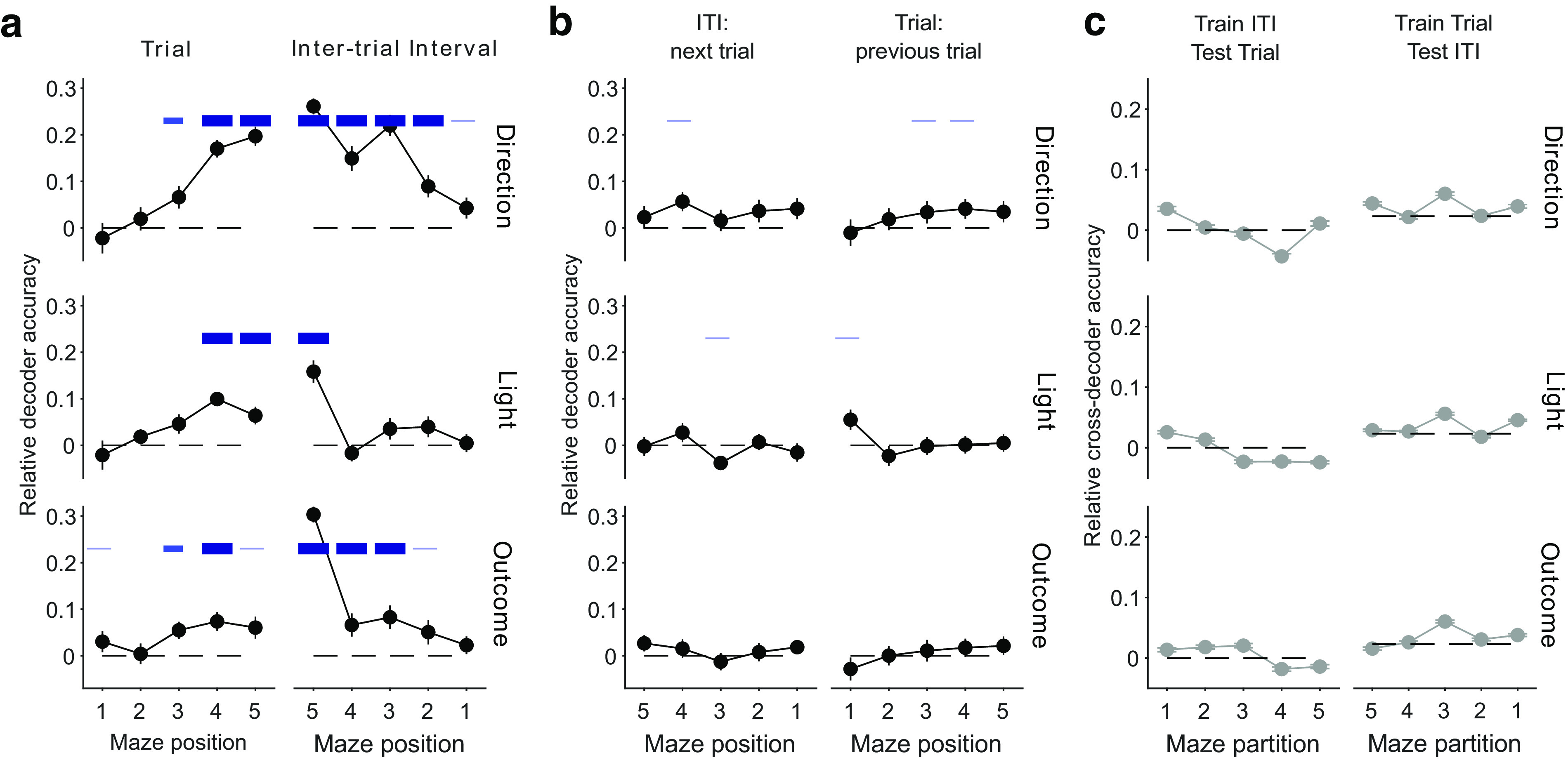
Population vector decoding across the maze. ***a***, Decoder performance at each maze location. We plot here decoding at consecutive locations across the trial and following ITI, with the decoding target for each location being the task feature in the current trial (so decoding the preceding trial during the intertrial interval). Blue bars give significance values of *p* < 0.05, thin blue line; *p* < 0.01, medium thickness blue line; *p* < 0.005, thick blue line; for Wilcoxon signed-rank test for difference from zero. Symbols plot means ± SEM across *n* = 49 sessions. ***b***, As per ***a***, for decoding the features of the following trial from the current intertrial interval (left) and decoding the features of the previous trial from the current trial (right). ***c***, Cross-decoding performance at each maze location is at chance.

**Figure 7.**
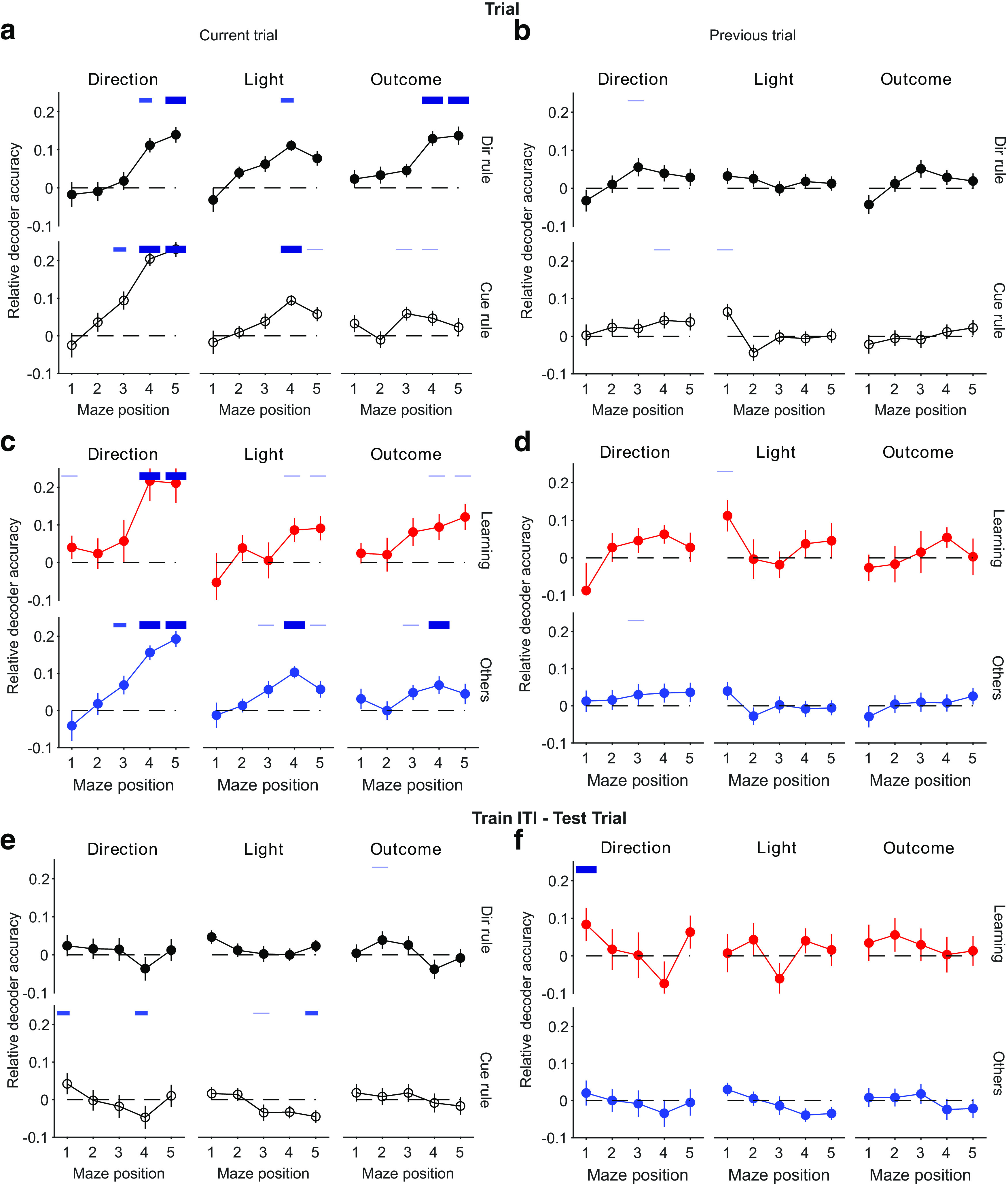
Robustness of location-dependent decoding in trials across session types. ***a***, ***b***, Breakdown of decoding performance in Figure 6*a*, according to the type of rule in each session, direction (15 sessions) or cued (34 sessions). ***c***, ***d***, Breakdown of decoding performance in Figure 6*a*, according to if the session contained putative rule learning (10 sessions, red symbols and lines) or not (39 sessions, blue symbols and lines). ***e***, ***f***, Breakdown of cross-decoding performance at each maze location (Figure 6*c*) by rule type (***e***) and performance type (***f***). Similar results were obtained for cross-decoding intertrial intervals from trials (data not shown).

Quoted measurement values are mean $\bar{x}$ and SEM. Differences between two paired distributions were assessed using a paired Wilcoxon signed-rank test; differences from zero were assessed using a Wilcoxon signed-rank test. In Figures 3-7 we report where *p* values for these tests exceeded three alpha levels (0.05, 0.01, 0.005). Differences between distributions were assessed using the Kolomogorov–Smirnov test. Throughout, we have *n* = 49 sessions; in some analyses we subdivide these into rule types (*n* = 15 direction-rule sessions and *n* = 34 cue-rule sessions) or learning types (*n* = 10 learning sessions and *n* = 39 Other sessions). In Figure 3 we break down the decoding results by each rat, giving *n* = 14, 14, 11, 14 sessions for rats 1–4, respectively.

### Data availability

The spike-train and behavioral data that support the findings of this study are available at http://crcns.org/data-sets/pfc/pfc-6.

## Results

We analyze here data from rats learning rules in a Y-maze that had tetrodes implanted in mPfC before the first session of training. Across sessions, animals were asked to learn one of four rules that were given in sequence (Go to the right arm, go to the lit arm, go to the left arm, go to the dark arm). Rules were switched after 10 correct choices (or 11 of 12). The animal self-initiated each trial by running along the central stem of the Y-maze and choosing one of the arms (Fig. 1*a*). The trial finished at the end of the arm, and a reward was delivered if the chosen arm matched the current rule being acquired. During the subsequent intertrial interval, the rat made a self-paced return to the start of the central arm to initiate the next trial. Trials were 6.5 ± 0.5 s on average; intertrial intervals were 55.6 ± 1.1 s. Throughout, population activity was recorded in the prelimbic and infralimbic cortex (Fig. 1*e*), for which we shall use the term mPfC here (Laubach et al., 2018, propose that these regions are equivalent to the anterior cingulate cortex in primates).

Neural activity statistics differed between trials and intertrial intervals. Neurons that had the highest firing rates in a trial tended to have lower firing rates in the following intertrial interval (Fig. 1*f*). However, the vector of rates across the population was not strongly correlated between the trial and following intertrial interval (Fig. 1*g*,*h*), meaning that changes in firing rates between the two phases of the task were not systematically in one direction. This low correlation of population activity between the trial and intertrial interval is also consistent with a change in representation as we now report.

### Separable subspaces of population activity between trials and intertrial intervals

We first asked whether population activity occupied different subspaces between consecutive pairs of trials and intertrial intervals as a basis for representing these as two different states of the world in the same population. To do so, we projected all population activity vectors of a session (Fig. 2*a*) into a low-dimensional space (Fig. 2*b*) and then quantified how easily we could separate them into trials and intertrial intervals. Using just one dimension for the projection was sufficient for near-perfect separation in many sessions; using two was sufficient for above-chance performance in all sessions (Fig. 2*c*). Population activity thus occupied a different low-dimensional subspace between the trials and the intertrial intervals.

This was not true within each phase (Fig. 2*d–f*): when we divided the maze into sections (Fig. 2*g*), the population activity at nearby maze positions was not easily separable within trials (Fig. 2*h*) or within intertrial intervals (Fig. 2*i*), with the notable exception of position 1—the return to the starting position—in the intertrial intervals. By contrast, population activity vectors at one position in the trial and another in the intertrial interval were easily separable between every pair of positions (Fig. 2*j*). Thus, although of course population activity changed across maze positions (Fig. 2*h*,*i*), those changes were smaller and continuous within each phase of the task but larger and discontinuous between them as they moved into a different subspace of activity.

A detailed examination of what might be driving this move into a different subspace of population activity is beyond our scope here, but we can show there are at least two plausible causes. Aligning population activity to the trial and intertrial interval implies this change is caused by reaching the arm end. But a range of other salient events may be causing this shift in population activity. To begin examining possible events, we instead divided the task into inbound and outbound phases, where the start of the outbound phase was defined from the point where the heading direction of the animal had turned toward the start of the maze for at least 400 ms, which happened on average 1.14 s later than the start of the intertrial interval (Fig. 2*k*). Dividing the population activity accordingly (Fig. 2*l*), we found the separability of inbound and outbound phase population vectors was excellent even in only a few dimensions (Fig. 2*m*,*n*).

Indeed, population separability was equally good for both the trial-ITI and inbound-outbound separations (Fig. 2*o*). It is thus equally plausible that the shift of subspace occupied by population activity is driven by a change in heading direction as by reaching the arm end. As these events occur close together in time (Fig. 2*k*), considerable further work, and likely additional experiments, would be necessary to tease apart the causal mechanisms (see Discussion). For the remainder of the article we thus continue to consider activity subspaces defined by the trial-ITI split while being mindful that exactly when the shift occurred and what causes it is unknown.

### Different states of the same task features can be decoded from population activity

We then tested whether these distinct subspaces corresponded to encoding different states of the world between the trials and intertrial intervals. Using a linear decoder on the vector of population activity during each trial (see above, Materials and Methods), we decoded key features of the task during the trial, that is, the choice of arm direction of the animal in the trial, the outcome of the trial, and which arm end was lit during the trial. We trained the same decoders using the same population vectors but with features shuffled across trials (see above, Materials and Methods) to define appropriate chance levels for each decoder given the unbalanced distribution of some task features such as outcome.

We could decode all direction choice, light position, and outcome in the current trial above chance (Fig. 3*a*,*b*, left). In Figure 3*a* we plot the absolute accuracy of decoding; in Figure 3*b* we also plot the decoding accuracy relative to the shuffled data for each session, which, as it accounts for the different distributions of features (e.g., outcome) in each session, better shows the effect size of the decoding. Relative decoding accuracies well above zero could even be seen for each animal (Fig. 3*d*), despite the small populations (Fig. 1*i*, median 10 neurons), the limited numbers of trials per session (median 29 trials) available for training the decoder, and the low number (11–14) of sessions. As the outcome was not yet known during the trial, the ability to decode outcome implies anticipatory activity for outcome in mPfC neurons, as previously reported by a number of labs (Euston et al., 2012). However, we found no correlation between the performance of an animal in a session and our ability to decode the upcoming outcome (Fig. 3*c*), suggesting this anticipatory activity is not dependent on how frequently reward was acquired. Nonetheless, although it is unclear what this anticipatory activity reflects, the ability to decode outcome was robust across all classifiers we tested (data not shown; see above, Materials and Methods; Maggi et al., 2018). To test for the effects of past states on population activity in the trials, we also tried decoding the direction choice, outcome, and light position of the preceding trial and found that decoding was at or close to chance (Fig. 3*a*,*b*, right; *e*, each subject). Population activity in mPfC during the trials thus depended on features in the present state of the task and weakly or not at all on features in the past trials.

In contrast, from population activity during the intertrial interval we could decode the direction choice, outcome, and light position of the immediately preceding trial well above chance (Fig. 3*f*,*g*, right), which could even be seen for each rat despite the relatively small number (11–14) of sessions each performed (Fig. 3*i*). One caveat is that although the light was extinguished during the intertrial interval, precisely when is not clear from the data we have available (see above, Materials and Methods); consequently, it is possible that the decoding of the light could represent in part its ongoing state. Decoding of the past outcome also did not depend on performance in the session (Fig. 3*h*). Decoding the same features of the immediately following trial was at chance (Fig. 3 *f*,*g*, left, *j*). Thus, trial and intertrial activity both represented distinct states of the world. Moreover, the evidence suggests trial activity represented the present, and intertrial activity predominantly represented the past.

### Independent decoding axes between the trials and intertrial intervals

Having found evidence that the activity of a single mPfC population occupies different subspaces encoding distinct states of the world, we could now ask whether and how the representations are kept distinct to downstream targets.

To compare the population coding between the trial and intertrial interval, we determined the decoding axis of trial activity for each of the present features and the decoding axis of intertrial interval activity for those same features in the preceding trial (see above, Materials and Methods). These decoding axes were close to orthogonal for all three features: the angles cluster at or close to π/2 (or, equivalently, their dot-product clusters at or around zero; Fig. 4*a*). And although the decoding axes for direction choice and light position departed from purely orthogonal, the median departure was small, being 0.067π for direction and 0.045π for light position. These differences between trial and intertrial decoding axes were also consistently and substantially larger than the differences within the same phase (Fig. 4*b*). Thus, the state of the world in the trial and intertrial interval can be independently decoded from the same mPfC population.

By contrast, within the trial and the intertrial interval, pairs of decoding axes for different features were not close to orthogonal, except the direction and outcome axes (Fig. 4*c*,*d*). This neatly demonstrates that the near orthogonality of the decoding axes between trials and intertrial intervals is not then a trivial consequence of the decoding axes being random vectors drawn from the same distribution because the decoding axes of the same dimension within each phase are not orthogonal. Notably, the distributions of angles between the decoding axes for a given pair of features were preserved between the trials and the intertrial intervals, with outcome direction around π/2, light direction centered below π/2, and light outcome centered above π/2. Thus, although each decoding axis rotated close to orthogonal between the trial and intertrial interval, the relationships between the feature decoding axes were preserved.

To quantify how distinct these independent axes made the decoding of the trial and intertrial states, we cross-decoded one from the other: for each feature type, we trained the classifier on all trials of a session and tested its ability to decode the same feature from the following intertrial intervals. We found that cross-decoding was at chance level for both outcome and light position, and significant but weak for direction (Fig. 4*e*), consistent with the angles between their decoding axes in the trials and intertrial intervals (Fig. 4*a*). This result was robust whether we trained on trials and tested on intertrial intervals or vice versa. Cross-decoding was also weak or at chance if we used leave-one-out testing instead (Fig. 4*f*) by leaving out the *i*th trial and its following intertrial interval, training on *N* – 1 trials, and predicting the *i*th intertrial interval. Thus the near independent decoding axes (Fig. 4*a*) indeed imply that downstream targets could independently read out either the trial or the intertrial state of the task from mPfC population activity.

### Decoding and cross-decoding are robust across types of session

We explored the extent to which this decoding depended on what occurred during each session. We first split the sessions by whether the target rule was direction based (15 sessions) or cue based (34 sessions). For trials, the present direction choice and outcome could still be significantly decoded for both types of rule, despite the considerable drop in power from the reduced number of sessions (Fig. 5*a*). For intertrial intervals, the preceding direction choice, outcome, and light position could still be decoded well above chance for both types of rule (Fig. 5*b*).

To determine whether learning itself affected any dependence on the task state, we then separated the sessions into two behavioral groups, putative learning sessions (*n* = 10), identified by a step change in task performance (see above, Materials and Methods), and the remaining sessions, called here Other (*n* = 39). We found decoding of task features was similar when comparing learning sessions and all Other sessions for both trials (Fig. 5*c*) and intertrial intervals (Fig. 5*d*). The sole exception, decoding the current light position during trials of Other sessions but not learning sessions, could be due either to a real effect or to the low power for decoding from 10 learning sessions.

For completeness, we also examined the breakdown of the cross-decoding results in Figure 4*d* by types of session. Figure 5, *e* and *f*, shows that cross-decoding of most features between trial and intertrial activity remained at chance, with again significant but weak cross-decoding of direction.

### Evolution of decoding within trials and intertrial intervals

It is likely that the decoding of task features from mPfC activity is partly dependent on maze position (Ito et al., 2015; Spellman et al., 2015). To further examine the evolution of decoding over the trial and intertrial interval, we again divided the maze into five equally sized sections (Fig. 2*g*) and constructed population firing rate vectors for each position. Although the trials averaged only ∼6.5 s in duration, and so each position was occupied for ∼1 s, we still obtained clear evidence for decoding the direction choice, outcome, and light position of the current trial across multiple contiguous locations (Fig. 6*a*, left). The contrast between the strong decoding of the features of the current trial and the weak decoding of the features of the previous trial was even clearer across maze positions (Fig. 6*b*, right).

This evolution means that there is contiguous decoding from the trial to the intertrial interval for all three features (Fig. 6*a*). Despite this contiguity, the cross-decoding between the same position in the two phases was at chance (Fig. 6*c*). In particular, cross-decoding at the arm end (position 5) was at chance, despite the rat continuously occupying this position during the transition from the trial to the intertrial interval. This suggests that the distinct decoding of the trial and intertrial states of the same feature appeared immediately at the arm end, or close to it (Fig. 2).

Figure 7 shows that these position-dependent decoding and cross-decoding results for trials are broadly robust to breaking them down by the type of rule or by learning behavior. Breakdowns of position decoding by session type in the intertrial intervals are given in Maggi et al. (2018), their Figure 5. In particular, we note here that the decoding of the state of the light during the intertrial interval only significantly occurs at position 5 when taken over all sessions (Fig. 6*a*, right), and as these data do not specify precisely when the light was extinguished during the interval, it is unknown whether that reflects the ongoing state of the light or the past state.

### Population representations of features reactivate in sleep

That the population activity occupies linearly separable subspaces between the trial and intertrial intervals (or the inbound and outbound phases) strongly suggests that the mPfC populations can be driven to either one or the other by upstream inputs. In turn, this implies that the representations of these two world states were independently addressable. To explore this question further, we turned to activity of the same populations during sleep.

Prior reports showed that patterns of mPfC population activity during training are preferentially repeated in post-training slow-wave sleep (Euston et al., 2007; Peyrache et al., 2009; Singh et al., 2019), consistent with a role in memory consolidation. However, these analyses looked only at specific templates or the reappearance of correlations between neurons, so it is unknown what task states these repeated patterns represented. Thus, we took advantage of the fact that our mPfC populations were also recorded during both pre-training and post-training sleep to ask whether their activity during sleep was specifically driven to either or both of the activity subspaces occupied by the population during the trials and intertrial intervals.

**Figure 8.**
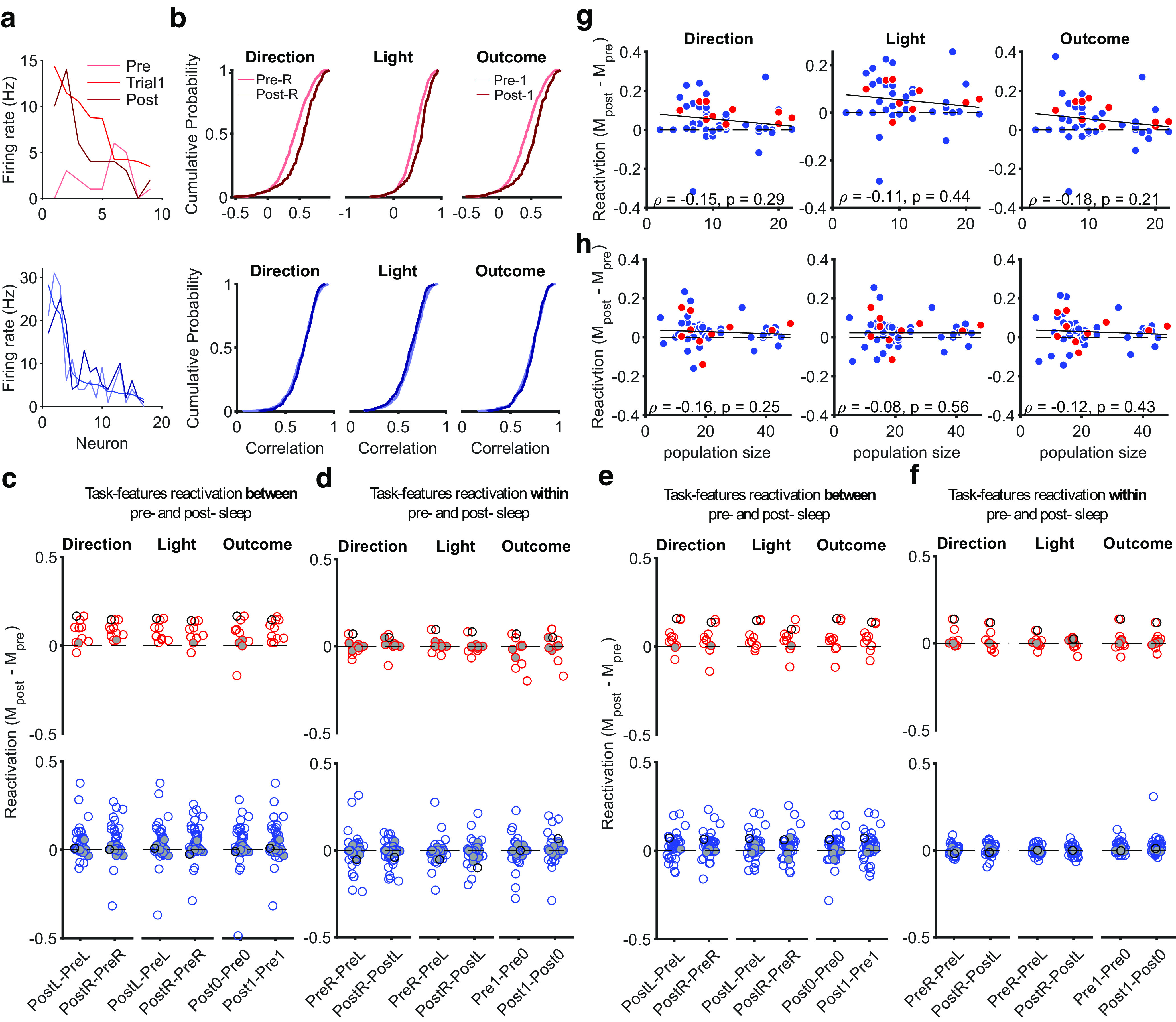
Reactivation of population subspaces in post-training sleep. ***a***, Example population activity vectors. Top, From one learning session, we plot the average firing rate vector for correct trials (Trial 1). For comparison, we also plot examples of firing rate vectors from pre-training and post-training slow-wave sleep (1 s bins). Neurons are ranked in order of their firing rates in the trial vector. Bottom, as for the top for an example session not classified as learning. ***b***, Example distributions of Spearman's rank correlations between trial and sleep population activity. Top, For the same learning session as ***a***, we plot the distributions of correlations between each vector of feature-specific trial activity and the population activity vectors in pre-training and post-training slow-wave sleep. Bottom, As for the top for the example nonlearning session in ***a***. R, Right arm; 1 indicates rewarded trial. ***c***, Summary of reactivations of trial activity across all sessions. For each task feature, we plot the difference between the medians of the pre-training and post-training correlation distributions. A difference greater than zero indicates greater correlation between activity in trials and post-training sleep. Each symbol is a session. Empty symbols are sessions with significantly different correlation distributions at *p* < 0.05 (Kolmogorov–Smirnov test). Gray filled symbols are not significantly different. One black circle for learning and one for nonlearning sessions identify the two example sessions in ***a*** and ***b***. ***d***, As for ***c***, but plotting the median differences between distributions for paired task features within the same sleep epoch. For example, in the far-left column, we plot the difference between the correlations with pre-training sleep activity for right-choice (PreR) and left-choice (PreL) specific trial vectors. ***e***, ***f***, As for ***c*** and ***d***, for intertrial interval activity. ***g***, Relationship between population size and the strength of preferential reactivation of trial activity. Symbols show sessions; red are learning, blue are Other sessions. Solid line gives the best-fit linear regression; text gives Spearman's rank correlation. ***h***, As for ***g***, for intertrial interval activity.

We first tested whether population activity representing features in the trials reactivated during slow-wave sleep. For each feature of the task happening in the present (e.g., choosing the left arm), we created the mean vector of population activity specific to that feature during trials in a session. This average population vector thus represented the region of the activity subspace (Fig. 2) occupied during trials with that feature. To seek reactivation of this region of the subspace in slow-wave sleep, we computed population firing rate vectors in pre-training and post-training slow-wave sleep in time bins of 1 s duration and correlated each sleep vector with the feature-specific trial vector (Fig. 8*a*). We thus obtained a distribution of correlations between the trial vector and all pre-training sleep vectors and a similar distribution between the trial vector and all post-training sleep vectors. Greater correlation with post-training sleep activity would then be evidence of preferential reactivation of feature-specific activity in post-training sleep.

We examined reactivation separately between learning and Other sessions, seeking consistency with previous reports that reactivation of waking population activity in mPfC most clearly occurs immediately after rule acquisition (Peyrache et al., 2009; Singh et al., 2019). Figure 8*b* (top) shows an example of a learning session with preferential reactivation. For all trial features, the distribution of correlations between the trial and post-training sleep population activity is right shifted from the distribution for pre-training sleep. For example, the population activity vector for choosing the right arm is more correlated with activity vectors in post-training (PostR) than pre-training (PreR) sleep.

Such post-training reactivation was not inevitable. In Figure 8*b* (bottom), we plot another example in which the trial-activity vector equally correlates with population activity in pre-training and post-training sleep. Although specific pairs of features (such as the left and right light positions) differed in their overall correlation between sleep and trial activity, no feature shows preferential reactivation in post-training sleep.

These examples were recapitulated across the data (Fig. 8*c*). In learning sessions, feature-specific activity vectors were consistently more correlated with activity in post-training than pre-training sleep. By contrast, the Other sessions showed less consistent preferential reactivation of any feature-specific activity vector in post-training sleep. As a control for statistical artifacts in our reactivation analysis, we looked for differences in reactivation between paired features (e.g., left vs right arm choice) within the same sleep epoch and found these all center on zero (Fig. 8*d*). Thus, population representations of present task features in the trials were preferentially reactivated in post-training sleep, and this most consistently occurred after a learning session.

We repeated the same analyses using feature-specific population vectors from the intertrial interval activity and also found evidence of preferential reactivation in some sessions (Fig. 8*e*,*f*). However, in contrast to trial activity, there was no consistent preferential reactivation of intertrial interval activity after a learning session.

Neither the preferential reactivation of trial nor intertrial activity was explained by significantly higher correlations between waking and sleep activity vectors from smaller populations (Fig. 8*g*,*h*).

**Figure 9.**
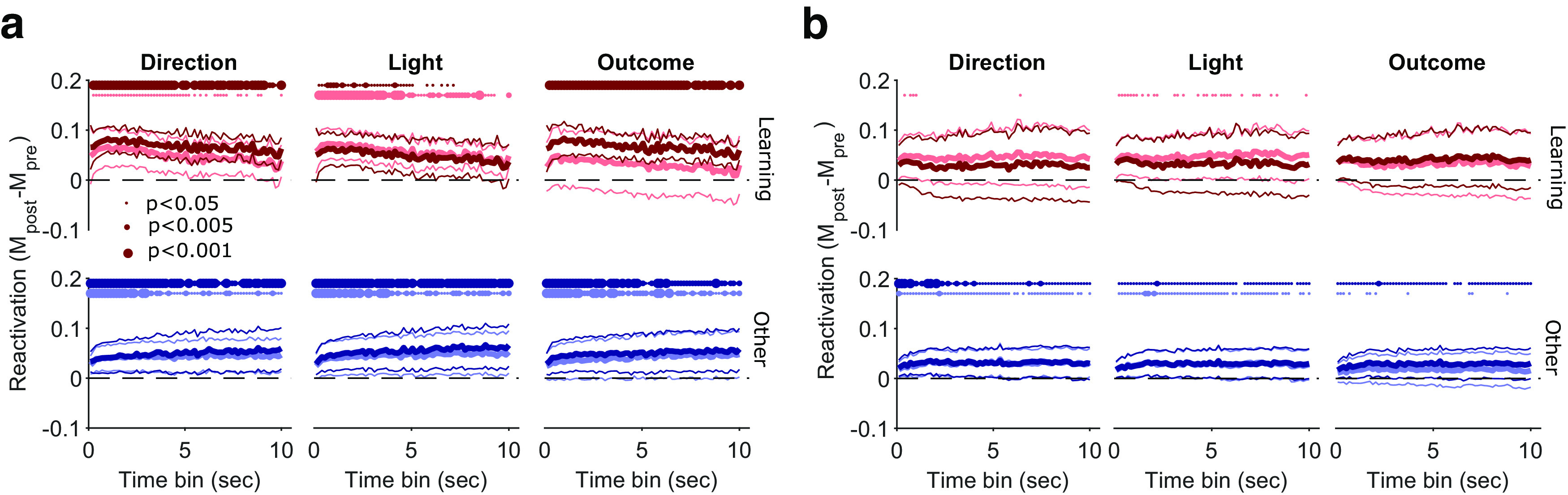
Robust preferential reactivation across time scales of sleep activity. ***a***, Preferential reactivation of trial activity across time scales. At each time bin used to construct population activity vectors in sleep, we plot the distribution over sessions of the median differences between pre-training and post-training correlation distributions for learning (top), and other (bottom) sessions. Distributions are plotted as the mean (thick lines) ± 2 SEM (thin lines); at the 1 s bin, these summarize the distributions shown in full in Figure 8*c*. Left, middle, and right columns plot two distributions, one per pair of features; lighter colors indicate left (L) or error trials (0), whereas darker colors indicate right (R) or correct trials (1). Time bins range from 100 ms to 10 s, tested every 150 ms. Dotted lines at the top of each panel indicate bins with reactivation significantly above zero (Wilcoxon signed-rank test, *p* < 0.05 small dot; *p* < 0.005 midsize dot; *p* < 0.001 large dot; *N* = 10 learning, *N* = 39 Other sessions). ***b***, As for ***a***, for intertrial activity.

As our measure of reactivation is asking whether and when the activity of the mPfC population revisits the trial and/or intertrial activity subspaces, it could do so on a range of time scales. These patterns of preferential reactivation were consistent across a range of bin sizes used to construct the activity vectors during sleep (Fig. 9). Notably, across these time scales, trial activity showed two independent properties from intertrial interval activity—consistent preferential reactivation after learning sessions, and preferential reactivation in those sessions was stronger at smaller bin sizes. These results are consistent with trial and intertrial activity subspaces being independently addressable; we thus sought further evidence of their independence.

### Independent properties of trial and intertrial activity reactivation in sleep

**Figure 10.**
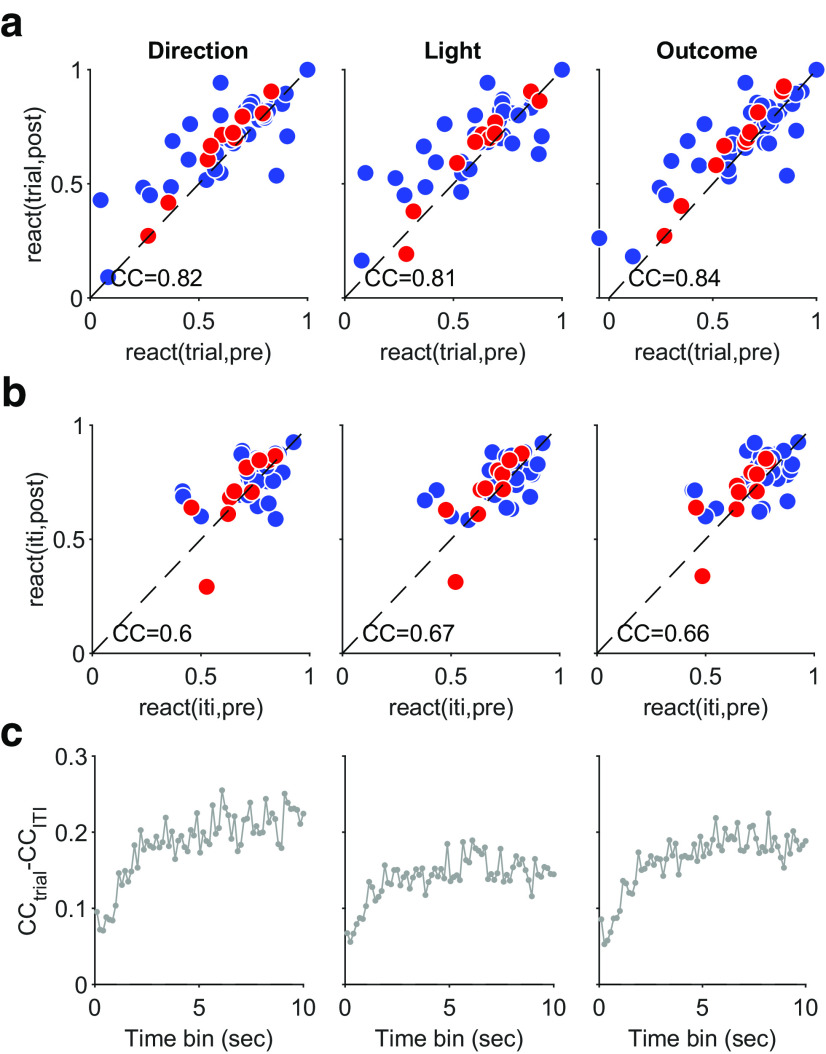
Trial activity reactivation is more correlated between pre-training and post-training sleep. ***a***, Correlation between the reactivation of trial activity in pre-training and post-training sleep for each task feature. Reactivation is as defined in Figure 8*a*, the median rank correlation between the waking and sleep activity vectors. Note the preferential reactivation is shown by symbols falling above the dashed identity line. Sleep activity vectors constructed using 1 s bins. CC, Correlation coefficient. Red indicates learning, blue indicates Other. ***b***, As for ***a***, for intertrial activity. ***c***, Difference between trial and intertrial activity reactivation correlations across bin sizes used to construct sleep activity vectors.

We asked whether the amount of reactivation of population activity differed between trial and intertrial activity. The reactivation of trial population activity was strongly correlated between pre-training and post-training sleep (Fig. 10*a*), but the reactivation of intertrial interval activity was less correlated (Fig. 10*b*), and this was consistent across time scales used to construct the sleep activity vectors (Fig. 10*c*). Thus the overall reactivation of trial and intertrial interval activity was consistently different, again suggestive that the two subspaces of activity were independently addressable.

**Figure 11.**
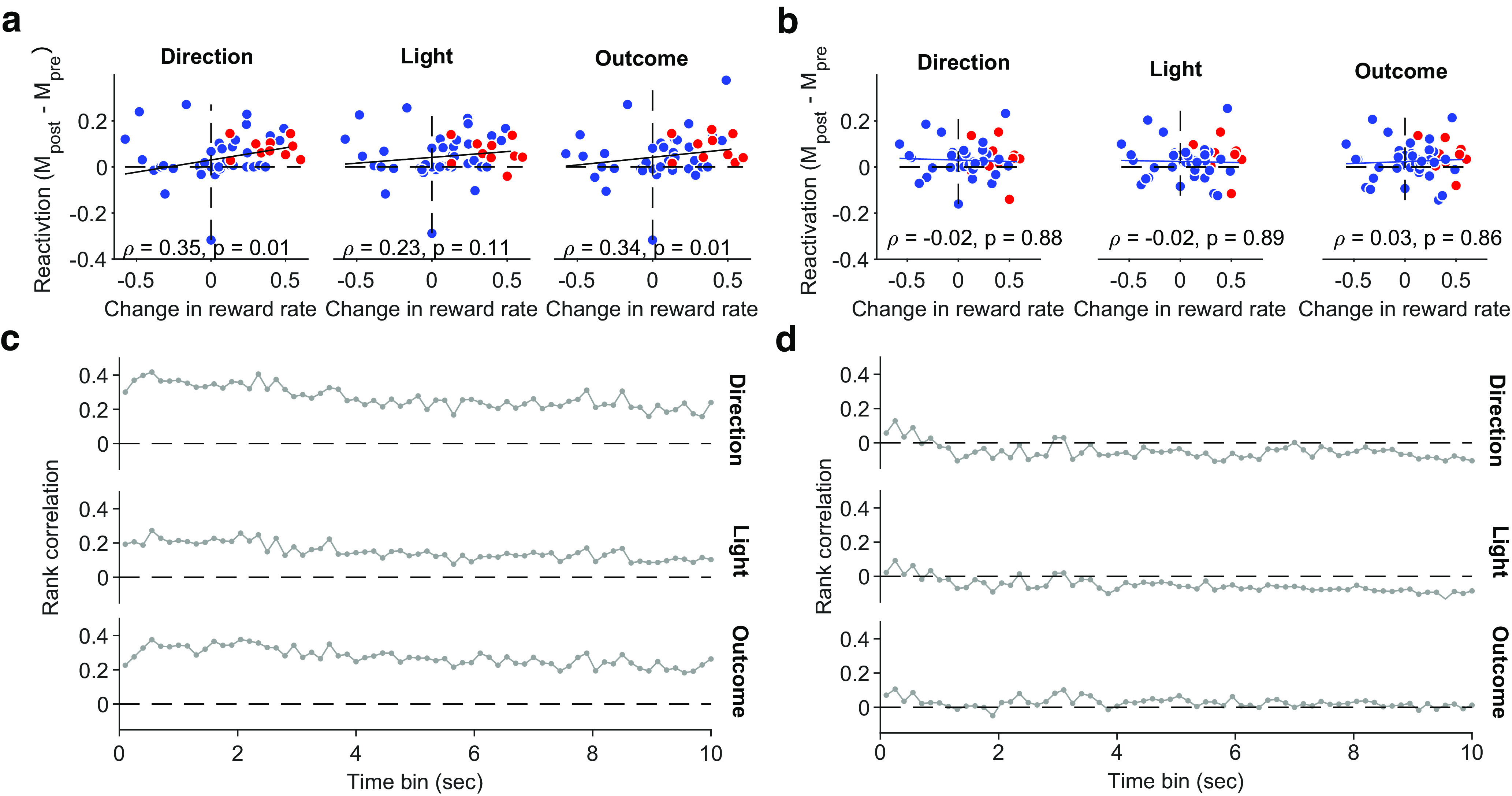
Preferential reactivation of trial activity correlates with performance. ***a***, Correlation between performance and preferential reactivation of trial activity. Sleep activity vectors constructed using 1 s bins. Values are Spearman's rank correlations. We plot here reactivation of vectors corresponding to left (direction and light) or correct; correlations for other feature-specific vectors are similar in magnitude, 0.37 (choose right), 0.35 (cue on right), 0.2 (error trials). ***b***, As for ***a***, for intertrial interval activity. Correlations for other feature-specific vectors are similar in magnitude, −0.004 (choose right), 0.02 (cue on right), −0.08 (error trials). ***c***, Correlations between performance and preferential reactivation of trial activity across bin sizes used to construct sleep activity vectors for the features in ***a***. ***d***, As for ***c***, for intertrial interval activity.

Given the above evidence that reactivation of trial and intertrial interval activity could be independently controlled, we further asked whether they differed in how preferential reactivation correlated with behavior. Following the differences in reactivation after learning sessions (Fig. 9), we looked at the degree of learning in a session, which we quantified by the size of the change in reward rate in that session (see above, Materials and Methods). We found preferential reactivation of trial activity correlated with the change in reward rate (Fig. 11*a*), but preferential reactivation of intertrial activity did not (Fig. 11*b*). Again, this difference between trial and intertrial activity reactivation was consistent across a wide range of time scales used to construct the sleep activity vectors (Fig. 11*c*,*d*).

## Discussion

Activity in the prefrontal cortex is known to represent different states of the world, including the immediate past or present state in a range of tasks (Baeg et al., 2003; Averbeck et al., 2006; Fujisawa et al., 2008; Sul et al., 2010; Rigotti et al., 2013; Hanks et al., 2015; Ito et al., 2015; Siegel et al., 2015; Spellman et al., 2015; Guise and Shapiro, 2017). How the representations of the different states relate to each other, and whether they coexist in the same population of neurons, has been unclear. Consequently, it is unknown how downstream readouts of prefrontal cortex activity can distinguish activity representing different states of the world.

Here, we have shown one potential solution in the medial prefrontal cortex of rats learning rules in a Y-maze: different states are encoded in the same population in such a way that linear decoders can read out different states of multiple features of the task. That encoding had two notable features. First, population activity is linearly separable between the trial and intertrial interval in as little as one dimension and so exists in different subspaces during these two phases of the task. Second, the decoding was roughly orthogonal between the trial and intertrial activity. These two features allow a simple solution to the interference problem.

### The interference problem

Any neural population whose activity contains information about multiple states of the world faces the problem of interference (Libby and Buschman, 2021), of how downstream populations can distinguish the activity that depends on each state, so that the sequence and causality of world events is clear. The inverse problem is how inputs to the population can selectively recall only the activity that depends on a particular state.

As we have shown here, because trial and intertrial activity occupies different subspaces of the population activity, a downstream target using a linear decoder can distinguish the two (Semedo et al., 2019). This suggests a simple solution to the interference problem of having two downstream populations, one whose input weights from the mPfC population match the decoding axis for the trial state and another whose input weights from the mPfC population match the decoding axis for the intertrial state. Then the first downstream population only responds to activity representing the state of the trial and the other only to activity representing the state of the intertrial.

**Figure 12.**
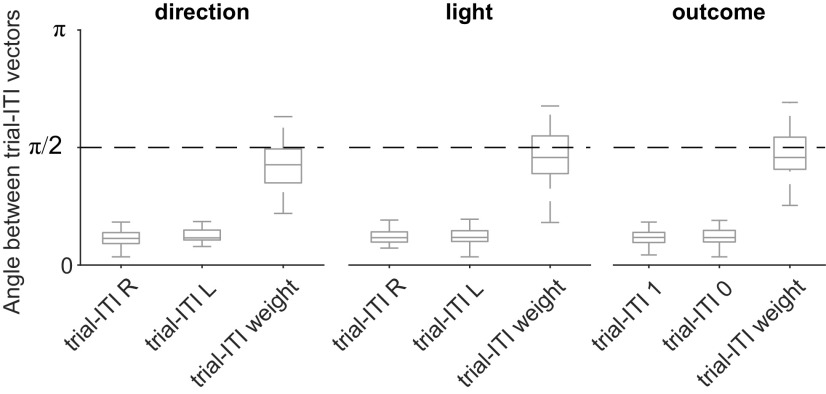
Population activity is not orthogonal, only decoding axes. For each feature (e.g., choose left), we plot the distribution of angles between the corresponding mean activity vector in trials and intertrial intervals. We also replot from Figure 4*a* the distribution of angles between the trial and intertrial interval decoding axes for that feature. L, Left; R, right. The number 1 indicates reward, 0 indicates no reward.

Key here is that the decoding axes are orthogonal, or close to it, although the population activity in mPfC is not. In Figure 12 we show this by plotting the angles between the mean activity vectors representing each feature in trials and intertrial intervals: we see that the activity representing each feature is more closely aligned between trials and intertrial intervals than are the corresponding decoding axes. Despite this alignment, because the activity sits in different linearly separable subspaces between trials and intertrial intervals, the different states of the task in the trial and in the intertrial interval are easily distinguishable by a linear decoder.

We also found evidence here of a solution to the inverse problem, as the existence of different subspaces of activity between trials and intertrial intervals means that upstream inputs could in principle separately drive the population activity to either subspace. The sequential decoding also strongly suggests that the same mPfC population can be driven into different representations by upstream inputs.

To explore this further we looked at activity of the same mPfC populations during sleep to ask whether trial and intertrial intervals representations of the task features are reactivated differently. Trial and intertrial activity were both preferentially reactivated in post-training slow-wave sleep, yet we found evidence that preferential reactivation of trial activity differed in the following four ways: the time scales at which it occurred most strongly, it occurred after learning sessions, the strength of reactivation was more consistent between pre-training and post-training sleep, and it correlated with the performance of the rats in the sessions. Together, these differences between the reactivation of trial and intertrial interval activity are consistent with upstream inputs to the mPfC population being able to separately address the representations of these states.

The consistency of preferential reactivation across broad time scales suggests that it is the changes to the relative excitability of neurons within the mPfC population that are carried forward into sleep (Singh et al., 2019). Thus, this consistency across broad time scales implies that whenever the neurons of the population are active, they are active together with approximately the same ordering of firing rates.

### Mixed population coding in mPfC

Our finding that small mPfC populations can sustain mixed encoding of two or more of direction choice, light position, and outcome of the current trial is consistent with prior reports of mixed or multiplexed coding by single neurons in the prefrontal cortex (Jung et al., 1998; Horst and Laubach, 2012; Rigotti et al., 2013; Fusi et al., 2016; Aoi et al., 2020). These encodings were also position dependent. Decoding of direction choice reliably occurred from the choice point of the maze onward, but it is unclear whether this represents a causal role in the choice itself or an ongoing representation of a choice being made.

Indeed, we are not claiming that the specific task features we decoded are necessarily explicitly represented in mPfC population activity. Rather, throughout we have interpreted the decoding of these features as evidence that mPfC population activity is at least representing the state of the world, similar to reinforcement learning views of PfC representations (Wang et al., 2018), because these features are a part of that state; and, hence, any change in one of those features, such as arm choice, would thus be a different state of the world.

Previous studies have reported that past choices modulated mPfC population activity during trials (Baeg et al., 2003; Sul et al., 2010). In contrast to the robust decoding of the present, we found weak evidence that mPfC activity during a trial depended on the light position of the previous trial and weak evidence that it depended on the direction choice of the previous trial only during direction-based rules. Moreover, these features of the past could only be decoded at one or two locations on the maze. Thus, during trials, population activity in the prefrontal cortex had robust, sustained dependence on multiple features of the present but at best weakly and transiently depended on one feature of the past.

Indeed, we have evidence here that the trial and intertrial activity represent not just different task states but, respectively, the present and past state of the task. Trial activity decoded present but not past features; intertrial activity decoded features of the preceding trial. The latter is consistent with well-established roles for the prefrontal cortex in short-term memory (Funahashi et al., 1989; Machens et al., 2010; Constantinidis et al., 2018; Lundqvist et al., 2018). However, the limitations of the Y-maze task data mean we cannot rule out that the intertrial activity also represented some features of the present during that interval, which is a question to be pursued further. Nonetheless, we have strong evidence that mPfC activity represents distinct states in the trials and in the intertrial intervals.

### What could drive changes in mPfC population activity

The evolution of activity within trials and intertrial intervals was continuous, with adjacent maze sections containing more similar population activity, yet the transition from the trial to the intertrial interval was discontinuous, with population activity moving to a different subspace, linearly separable from the trials' subspace. What might be driving this shift from the trial to the intertrial interval subspace of activity and hence its decodability?

The division into trials and intertrial intervals or the inbound and outbound phases in Figure 2 both distinguish the two legs of the journey in the maze. During the return trip to the starting position, the change in context and direction of movement would likely change the signals available to the mPfC. It does not automatically follow though that changes in context and movement cause the observed changes in population activity in mPfC; those changes to sensory and movement information could have changed mPfC population activity so that it did not encode anything about the immediately preceding trial, in the same way, for example, that we showed the intertrial activity encodes nothing about the immediately upcoming trial, even when the decision of the trial could be known in advance. Thus, our finding that we could still decode the state of the immediately preceding task features from intertrial activity despite the changes in context and movement information is nontrivial. Indeed, it implies that those changes could be the drivers of the observed changes in population activity.

This suggests multiple lines of fruitful further work here. One open question is which inputs to the mPfC drive the move from one activity subspace to another. Given the switch in context and heading direction, a likely candidate is the direct input from region CA1 of the hippocampus (Jones and Wilson, 2005; Benchenane et al., 2010, 2011). Another open question is precisely when the change in activity subspace happens. We showed here preliminary results that the larger, discontinuous change in population activity could be plausibly either on reaching the arm end or on initiating the outbound trip back to the starting position. Another is the precise function of the representations of the trial and intertrial interval; one possibility is they respectively reflect reward prediction and reward processing. One way to tackle this question would be to examine how much the clean independence between the decoding of task states depends on the behavioral task. For example, tasks where the future choice of arm depends on recent history, such as double-ended T-mazes (Jones and Wilson, 2005), multiarm sequence mazes (Poucet et al., 1991), or delayed nonmatch to place (Spellman et al., 2015), blur the separation of the present and the past. Comparing population-level decoding of the states in such tasks would give useful insights into when they are or are not independently coded within mPfC.
